# A theoretical quantitative genetic study of negative ecological interactions and extinction times in changing environments

**DOI:** 10.1186/1471-2148-8-119

**Published:** 2008-04-25

**Authors:** Adam G Jones

**Affiliations:** 1 Department of Biology, Texas A&M University, 3258 TAMU, College Station, TX 77843, USA

## Abstract

**Background:**

Rapid human-induced changes in the environment at local, regional and global scales appear to be contributing to population declines and extinctions, resulting in an unprecedented biodiversity crisis. Although in the short term populations can respond ecologically to environmental alterations, in the face of persistent change populations must evolve or become extinct. Existing models of evolution and extinction in changing environments focus only on single species, even though the dynamics of extinction almost certainly depend upon the nature of species interactions.

**Results:**

Here, I use a model of quantitative trait evolution in a two-species community to show that negative ecological interactions, such as predation and competition, can produce unexpected results regarding time to extinction. Under some circumstances, negative interactions can be expected to hasten the extinction of species declining in numbers. However, under other circumstances, negative interactions can actually increase times to extinction. This effect occurs across a wide range of parameter values and can be substantial, in some cases allowing a population to persist for 40 percent longer than it would in the absence of the species interaction.

**Conclusion:**

This theoretical study indicates that negative species interactions can have unexpected positive effects on times to extinction. Consequently, detailed studies of selection and demographics will be necessary to predict the consequences of species interactions in changing environments for any particular ecological community.

## Background

The growing human-induced extinction crisis [1-3] has added additional urgency to the development of theory related to extinction dynamics in natural populations. In the short term, some organisms can resist the effects of environmental change by behavioural changes, physiological acclimation, or migration [4]. However, in response to major, long-term environmental change, populations must evolve to avoid extinction [5-7]. Recent research showing that rapid evolution is common [8,9] and that it can affect the dynamics of species interactions [10] underscores the need to consider evolutionary responses to changing environments.

Theory concerned with the effects of evolutionary processes on community dynamics dates back several decades [11], but interest in eco-evolutionary dynamics has intensified dramatically over the last several years [12]. The results of these studies show that community dynamics certainly depend upon the details of evolutionary change in the interacting species. However, firm general results are difficult to obtain [12], as they depend upon a number of evolutionary parameters, such as details of inheritance and mutation, patterns of migration, and the nature of selection [12-14]. Thus, there is a need for more theoretical and especially empirical work. Furthermore, the implications of these models, which have focused mainly on static environments, with respect to extinction dynamics are not always transparent. For example, coevolution is expected to produce a geographic mosaic of maladaptation [14], and anthropogenic insults might be expected to disrupt coevolved ecological interactions [13]. Intuitively, these scenarios suggest that coevolution is a key factor in species persistence. However, it is not entirely clear under what circumstances coevolutionary dynamics increase or decrease probabilities of extinction. Because most studies of coevolution in ecological communities have focused on static environments [12], the study of coevolution in changing environments promises to produce interesting, complementary insights.

Several quantitative genetic studies of species persistence in changing environments have been conducted, but they did not consider species interactions. These single-species models of quantitative trait evolution and extinction show that a species' persistence time depends mainly upon the level of additive genetic variance for the ecologically relevant trait, the rate at which the environment changes, and the strength of selection [5,6]. The single-species models have been enlightening, but the dynamics of extinction in natural systems almost certainly depend on the nature of species interactions [15,16]. The single-species models cannot simply be extrapolated to predict how multi-species communities will respond to environmental change. This question calls for formal coevolutionary models. Thus, my goal was to extend single-species models of population persistence to communities consisting of two interacting species. Here I focus on negative ecological interactions, because *a priori *such interactions seem to have the most serious conservation implications [17,18]. In this brief report, I address one major question: What are the effects of negative ecological interactions on expected times to extinction in two-species communities evolving in response to a changing environment?

## Results and Discussion

**Figure 1:**
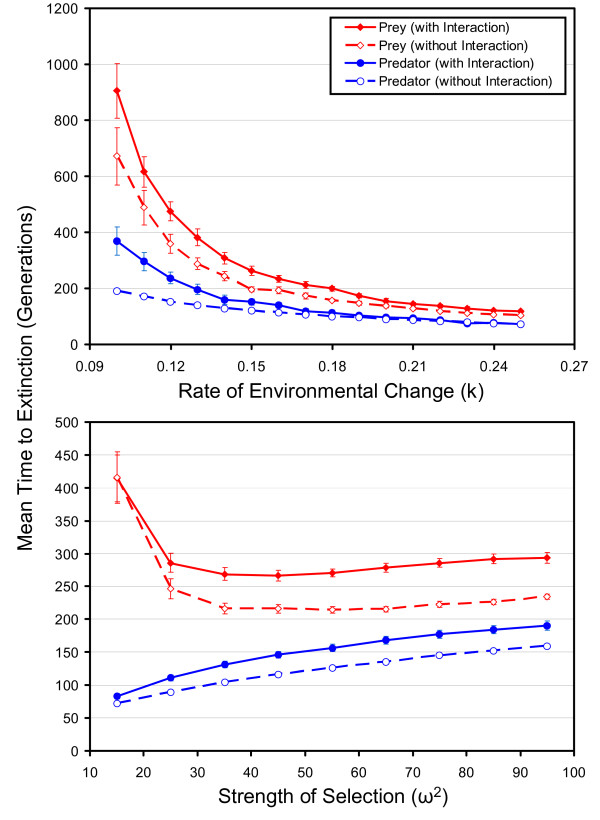
**The effects of a predator-prey interaction on mean extinction times of the two interacting species. **The top panel shows the effect under different rates of environmental change, whereas the bottom panel shows the effect for different strengths of selection. In both panels, extinction times of the prey are shown by the red symbols and lines, and those of the predator are shown by the blue symbols and lines. The solid symbols joined by solid lines represent results from experimental runs of the model with the predator-prey interaction intact, whereas the open symbols joined by broken lines show the control runs (in which the predator-prey interaction is removed). Over a wide range of rates of environmental change and strengths of selection, both the predator and prey persist longer when the two species interact than when they do not. Each point represents the mean of 40 runs of the simulation. In the top panel *ω*^2 ^is 49, and in the bottom panel k is 0.20. The values of *k *are in units of environmental standard deviations, which are slightly smaller than phenotypic standard deviations in this study. See methods for other parameters used to generate these data.

The results of the quantitative genetic model of species interactions and environmental change provide some insights into how negative ecological interactions might impact extinction dynamics. The most interesting result of this study is the counterintuitive observation that under a very wide range of conditions the presence of a negative ecological interaction actually increases the expected time to extinction for one or both species involved in the interaction. This effect is most pronounced in the predator-prey model. Figure 1 shows the mean persistence times of the predator and prey under different rates of environmental change and strengths of selection. When stabilizing selection is relatively weak (*ω*^2 ^= 49), a situation that is probably common in nature [19], the prey species always persists longer when it is preyed upon (Figure 1, upper panel). The time to extinction for the predator is also usually higher when it is interacting with the prey than when it is simply evolving in response to the moving optimum (Figure 1, upper panel). Only when the optimum is moving very quickly does the benefit of the interaction to the predator evaporate. Similarly, across a wide range of strengths of selection, both the predator and the prey benefit from their interaction (Figure 1, lower panel).

**Figure 2:**
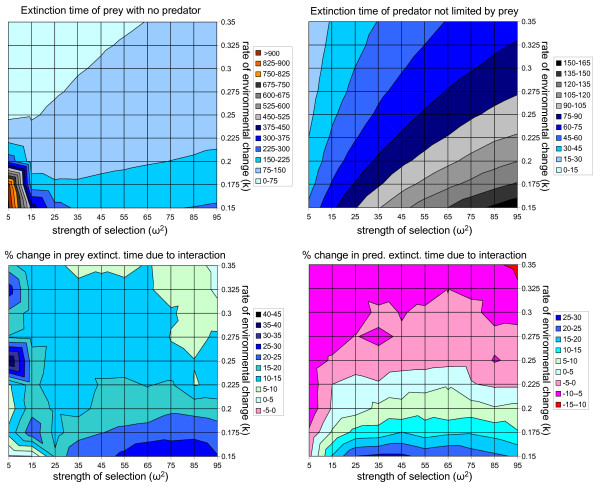
**An extensive exploration of the average extinction times in the predator-prey model under different rates of environmental change and strengths of selection.** The top panels show the average extinction times of the control populations (i.e., no interaction between predator and prey). The prey is on the left and the predator is on the right. The bottom panels show the percentage change in persistence times for the prey (left) and predator (right) when the predator-prey interaction is included in the model. Blue colors indicate combinations of parameters under which the population persisted longer due to the predator-prey interaction. See methods for the parameter values. I used values of *ω*^2 ^ranging from 5 to 95, in increments of 10, and values of *k *ranging from 0.15 to 0.35 in increments of 0.025. The values of *k *are units of environmental standard deviations for the traits, which are slightly smaller than phenotypic standard deviations in this study. For each combination of parameters, I averaged across 200 runs of the simulation to generate the means depicted in this figure.

Figure 2 shows a more extensive exploration of the effects of rates of environmental change and the strength of selection on mean extinction times in the predator-prey model. The prey almost always benefits from the predator-prey interaction, with benefits usually greater than 10 percent and sometimes as large as 40 percent or more (Figure 2, lower left panel). The rare exception to this pattern occurs when quadratic selection is very strong and the optimum is moving slowly. Interestingly, the predator also benefits from the interaction under a wide, but less extensive, combination of parameters (Figure 2, right panels). With an optimum moving at a slow or moderate pace, the predator's extinction time increases due to the predator-prey interaction (versus the control case in which all predator individuals survive without being required to catch prey). However, when the optimum moves at an extremely rapid pace (or the strength of selection is extremely strong), the expected time to extinction for the predator is shorter due to the predator-prey interaction. In these cases, the evolution of the prey outpaces the evolution of the predator, resulting in an insufficient number of susceptible prey individuals to sustain the predator population.

**Figure 3:**
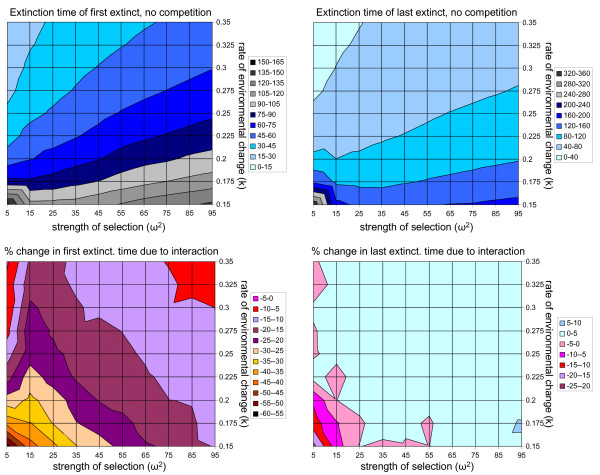
**Results of the competition model under different strengths of selection and rates of environmental change.** The top panels show average extinction times for the first species to go extinct (left) versus the last species to go extinct (right) with no interaction between the species. The bottom panels show the percentage change in expected extinction times due to the competitive interaction. Note that the first species to go extinct (which lags the greatest distance from the optimum) always goes extinct more rapidly when competition is present than when it is absent (bottom left). However, under most combinations of parameters, the species closest to the moving optimum persists for longer periods of time when competition is present than when it is absent (bottom right). See Methods and Figure 1 for parameter values.

Figure 3 shows the results of the competition model. Under all parameter values under consideration in this study, one of the species is at a greater risk of extinction due to the competitive interaction (Figure 3, lower left panel). Under a stationary optimum, competition results in character displacement, so when the optimum starts to move due to environmental change, one species starts out with a greater lag than the other. The species lagging farthest from the optimum always experiences a decrease in time to extinction due to competition, because the competitive interaction produces selection away from the optimum (i.e., maladaptation), which increases the lag relative to the case without competition. The species that starts closest to the optimum, however, often benefits from competition, because the selection induced by the moving optimum is augmented by selection in the same direction caused by competition, the net effect of which is a reduced lag and an increase in time to extinction. The positive effect in the competition model is more modest than the effect in the predator-prey model, but it occurs over a very wide range of parameter values (Figure 3, lower right). The only situation under which the species closest to the optimum experiences a major negative effect of competition occurs when selection on the trait responding to the moving optimum is very strong and the optimum is moving slowly.

**Figure 4:**
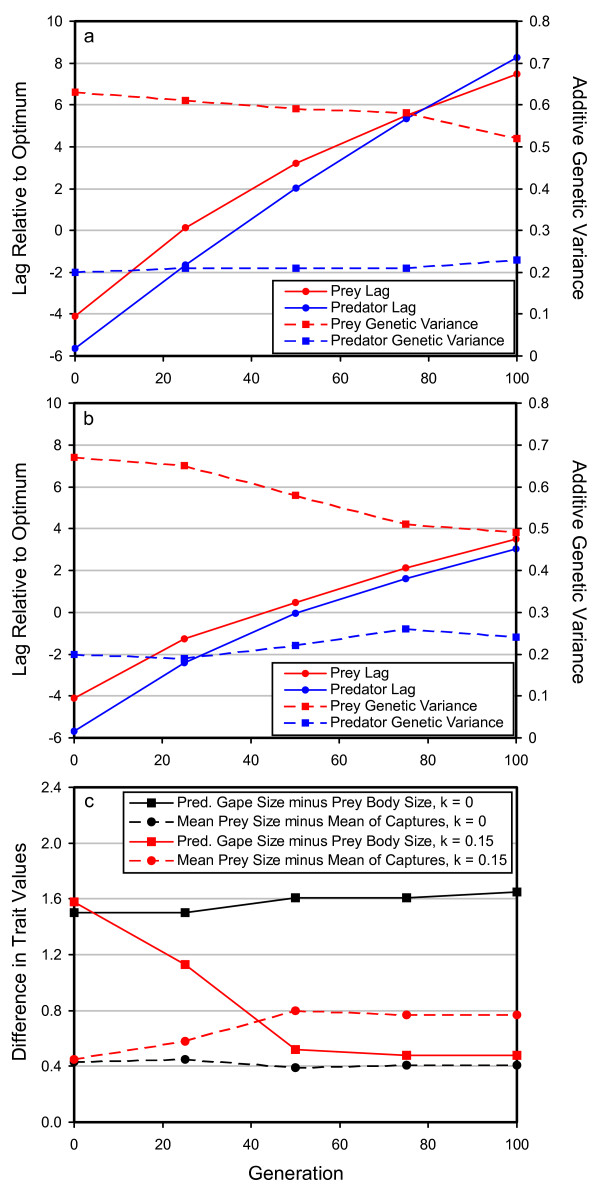
**Mechanistic details of the predator-prey model for a sample set of parameter values.** The top two panels show the lag and additive genetic variance over time in the predator-prey model for the two species in typical control (a) and experimental (b) runs of the simulation. When the predator-prey interaction is absent, the lag for both species increases rapidly (a). However, when the predator-prey interaction is present, the rate of increase of the lag is reduced for both species, permitting both species to persist for longer periods of time before extinction (b). Under these parameter combinations, the reduced lag occurs for both the predator (solid blue lines) and the prey (solid red lines). The additive genetic variances of the predator (broken blue lines) and prey (broken red lines) do not differ dramatically between the experimental and control runs, except that there is a slightly more rapid loss of genetic variance in the prey during the experimental runs. The bottom panel (c) shows the tendency for the predator to eat the most maladapted individuals as the optimum moves. When the optimum does not move (black lines), the difference between mean predator gape size and mean prey body size reaches a steady-state expected value. A similar situation occurs for the difference between the mean size of prey in the population and the mean size of prey that are actually eaten. As the optimum moves (red lines), however, the gape size of the predator decreases relative to the mean prey size, and the difference in size between mean prey size and predated individuals becomes even greater than it is in the population experiencing a stationary optimum. The results depicted in this figure used the standard set of parameter values (see methods), and *k *and *ω*^2 ^were set to 0.15 and 50, respectively. Each point on each graph is a mean from 50 replicate runs of the simulation.

The cause of the increase in expected times to extinction can be seen by examining some of the mechanistic details of the model. Figure 4 shows the dynamics of lag of the phenotypic mean relative to the optimum in control (Figure 4a) and experimental (Figure 4b) runs of the predator-prey model. The most striking result is that the lag increases much more rapidly in the control run (with no species interaction) than in the experimental run (with a predator-prey interaction). The reason that the presence of a predator-prey interaction decreases the lag is that the predator culls the individuals in the population that are least well adapted to the new phenotypic optimum, because they are closest in phenotype to the individuals that the predator evolved to prey upon. Effectively, predation increases the strength of selection on the phenotype and pushes the phenotypic mean of the prey to keep up with the moving optimum. The predator similarly benefits, because (assuming that the predator's optimum is also moving) the predators that are closest to the moving optimum will also be the most effective predators on the evolving prey. The predators far from the moving optimum will be poor predators and will fail to catch enough prey to reproduce. Thus, the presence of an evolving prey population also increases the intensity of selection on the predator in the direction of the moving optimum. A similar explanation applies for the competition model. The competitor lagging least far behind the optimum is pushed to keep up with the optimum by its competitor, because competition tends to kill off the individuals that are least well adapted to the new optimum each generation. This phenomenon obviously decreases the lag, which increases the time to extinction.

Figure 4 also shows that the positive effects of species interactions on extinction times occur mainly as a consequence of the culling of maladapted individuals, rather than as an outcome of any major effects on the standing genetic variance for the evolving trait. The prey in the predator-prey model experiences a gradual decline in additive genetic variance as a consequence of the moving optimum, and the decline is slightly more rapid in experimental runs (Figure 4b) than it is in control runs (Figure 4a), but otherwise the control runs look very similar to the experimental runs with respect to the dynamics of the additive genetic variance.

The final mechanistic detail of interest, illustrated by Figure 4c, is that the tendency for the predators to eat the least well-adapted individuals is an intrinsic evolutionary outcome of a predator-prey system responding to a moving optimum. This model uses a gape-limited predator, so predator gape size evolves to be larger than the mean prey body size in the absence of a moving optimum. As the optimum begins to move (in a positive direction) the mean predator gape size lags behind the mean prey body size, and consequently the predators prey more heavily on the smaller, maladapted individuals. Regardless of the exact details of the predator-prey interaction (i.e., gape-limited or otherwise), predators generally should evolve to prey upon individuals near the phenotypic mean of the prey species in an unchanging environment [20]. Hence, a move of the prey optimum, coupled with the lag of the predator's phenotype relative the prey's phenotype, should typically result in a situation in which predators prey upon maladapted individuals in changing environments.

Parameters other than the strength of selection and the rate of environmental change also affect mean extinction times. For example, the mutation rate, the distribution of allelic effects, the carrying capacity, and the birth rate can affect times to extinction in this model. However, the effects of these parameters have been examined in detail in the single-species model [6], and the conclusions of the single-species model also apply to the two-species models that I investigated. For instance, larger carrying capacities and birth rates increase expected times to extinction in the multi-species model as they do in the single-species model. Regardless of the exact values of these parameters, however, predation and competition still produce a positive effect on extinction times for one or both species involved in the species interaction under many rates of environmental change and strengths of selection. Thus, the result that negative ecological interactions often increase times to extinction appears to be robust to changes in most of the parameters of the model.

While additional work on the phenomena documented here is warranted, this study does carry potential management implications. For example, one possible reaction to a predator preying upon a species at risk of extinction might be to somehow protect the prey from the predator. Whether or not this move would benefit the species of interest depends upon the consequences of the species interaction. In a changing environment, if the predator removes individuals closer to the moving optimum relative to the mean phenotype, then the predator is harming the species of concern. However, if the predator is removing maladapted individuals, then predation may actually be delaying extinction of the declining species. Similar arguments apply for competitive species interactions. Thus, a complete management strategy should attempt to model the demographic and evolutionary effects of species interactions with models parameterized for the species of interest.

Both competition and predation of the type considered in this study appear to occur with high enough frequency in natural populations to be important. In addition, the generality of the phenomenon observed in this model implies that other types of negative ecological interactions will likely produce the same positive results on species persistence times. Several important studies have found evidence for reciprocal selection in predator-prey systems. For example, red crossbills exert a directional selective pressure on lodgepole pine, while lodgepole pine cone shape causes stabilizing selection on bill size [21]. In addition, the classic example of toxic newts and their garter snake predators represents another system that appears to have the necessary elements for predator-prey coevolution [22]. In either of these cases, evolution of the prey (due to environmental change) would result in selection on the predator and could produce the type of situation that occurs in the present model. Similar examples of competition appear in the literature. For example, competition for seeds in Darwin's finches is analogous to the type of competition that I model here. Coexisting species exhibit ecological character displacement for beak characteristics [23], and changes in the distribution of seeds in the environment results in strong directional selection on the finches [24]. The prediction of the present model would be that adaptation of the species with beak characteristics best matched to the changing food supply would be facilitated by the presence of a less well adapted competitor.

My analysis is an initial attempt to address the effects of species interactions on extinction times in changing environments, but it raises a number of questions that would be worthy of additional research. The most limiting assumption of the current study is that the same trait that mediates the species interaction also changes in response to the environment. Future work should address cases in which the trait involved in the species interaction is distinct, but possibly genetically correlated, with the trait responding to environmental change. The present study would then be a special case in which the genetic correlation is unity. Thus, with high genetic correlations among traits, results would be similar to those reported here. However, the dynamics of systems with weak or negative correlations among traits would certainly be of interest.

Another possible limitation of the current analysis concerns the assumption that the optimum moves unidirectionally at a constant rate. This limitation could be partially corrected by including stochasticity in the movement of the optimum, a situation that would almost certainly decrease mean species persistence times [6]. However, the effects of the species interaction would likely still be present in such a model. Future work should consider species interactions in multivariate models in which the optima of suites of traits move in response to environmental change. The challenge in such models will be to relate theoretical models of the movement of the multivariate optimum to realistic expectations for actual organisms. This latter point brings up another limitation of the present study, which is that it is entirely theoretical. Future work should apply similar models to real negative species interactions that have been studied sufficiently to produce empirical estimates of parameter values for the model. Before generalities regarding management practices can be drawn, some real case studies should be investigated in detail.

## Conclusion

If the generality of this model can be confirmed with additional research and empirical examples, then the phenomena documented here may be important from a conservation standpoint. One important result is that it should be possible to determine empirically which negative species interactions should be retained and which should be halted in particular managed ecological communities. If the predator or competitor is causing the deaths of individuals that are poorly adapted to the changing environment, then the species interaction probably will facilitate adaptation. On the other hand, if the species interaction is causing the demise of the individuals that are best adapted to the new environment, then the species interaction will hinder adaptation and should be dealt with from a management standpoint. Even though this model only applies to changing environments, it is important to keep in mind that the major conservation concern of our generation, anthropogenic extirpation of species, is by definition caused by human-induced environmental change, so the findings of this model are likely to be relevant for a broad spectrum of conservation crises. The bottom line is that this preliminary analysis clearly shows that even so-called "negative" species interactions need to be carefully examined from a management and evolutionary standpoint.

## Methods

### The quantitative genetic model

The model is an elaboration of the one used by Bürger and Lynch [6] to study the response of a single species to environmental change. The major change is that the new model is coevolutionary, because it simultaneously follows the evolution of two populations of organisms that interact via predation or competition. The model is a Monte Carlo simulation of all individuals in two distinct populations reproductively isolated from one another but coexisting. Consequently, a response to selection in one species changes the selection regime in the other species, resulting in reciprocal evolutionary change. I assume additive genetic effects and explicitly model all genetic loci within all individuals in each species. The life cycle consists of (1) production of offspring, including free recombination and mutation, (2) directional and stabilizing selection specified by the curvature of the individual selection surface, the position of the optimum, and the details of the ecological interaction, and (3) random choice of *K *adults from the survivors of selection to make up the next generation, where *K *is the carrying capacity. If fewer than *K *individuals survive selection, then all of the survivors are allowed to contribute to the next generation of progeny.

Each population is characterized by a single quantitative trait determined by *n *unlinked loci and subject to stabilizing and directional selection. Mutations occur at a rate of *μ *per locus per gamete and new mutational effects are drawn from a Gaussian distribution with a variance of *α*^2 ^and a mean of zero according to the continuum-of-alleles model [25]. An individual's phenotypic value is determined by summing across loci and adding environmental variance drawn from a normal distribution with a mean of 0 and variance of 1. The probability of surviving selection imposed by the environment in a particular generation is

(1) W_z,t _= exp[-0.5(*z *- *θ*_*t*_)^2^/*ω*^2^],

where *z *is the individual's phenotypic value, *θ*_*t *_is the optimum at time *t*, and *ω*^2 ^is the width of the individual selection surface. The mating system is monogamous, and each reproducing pair produces exactly 2*B *offspring. Most parameter values are set at the values used by previous studies that have employed this type of model [6,26,27]. Specifically, I use *n *= 50, *μ *= 0.0002, and *α*^2 ^= 0.05. Environmental change is included in the model by allowing the phenotypic optimum to move each generation, according to the relationship

(2) *θ*_*t *_= *kt*,

where *θ*_*t *_is the position of the optimum at time *t *and *k *is the per generation rate at which the optimum moves. In the single-species case, selection causes the mean phenotype to track this moving optimum but lag behind it [5,6].

Each run of the simulation began with 5,000 generations of evolution according to stabilizing selection, during which an initially genetically uniform population reached a mutation-drift-selection equilibrium. These generations were followed by 1,000 generations during which the species interacted (see below for details of species interactions) in the absence of environmental change (i.e., k = 0) to allow the interacting species to reach a quasi-equilibrium. I only investigated parameter combinations that allowed the two species to coexist under a stationary optimum. The 1,000 initial generations of interaction were followed by up to 100,000 experimental generations during which the optimum for one or both species was allowed to move, while all other parameters governing the ecological interaction remained unchanged. The main response variable of interest was the time to extinction, so the experimental generations ended when both species went extinct.

Control runs were exactly the same as experimental runs, except that the ecological interaction was removed at the beginning of the experimental generations. Thus, the control runs began with the same expected phenotypic distributions and levels of genetic variance as the experimental runs. This control is the most appropriate for this study, because it allows a rigorous test of the effects of the ecological interactions *per se *on extinction times, while controlling for the effects that the ecological interaction has on the phenotypic and genetic characteristics of the population at the beginning of the experimental generations. The choice of parameter combinations was guided by the single-species results of [6].

### The predator-prey model

The easiest way to envision the predator-prey model is as a potentially gape-limited predator and its prey, although this model (or slight variations of it) can apply to other types of predator-prey interactions. Thus, the trait in the predator is gape size and the trait in the prey is body size. The survivors of viability selection (see above) were allowed to encounter *N*_*ENC *_prey per predator at random, with a probability *P*_*C *_of catching each prey item encountered that was smaller than its gape and a probability of zero of capturing prey items larger than its gape. A predator was required to capture at least *N*_*MIN *_prey to survive. This predator-prey model is similar to other models that have been developed to study the evolution of quantitative traits in predator-prey interactions (reviewed by [20]). Alternative versions of the model, including density dependent prey encounter rates and a positive relationship between number of prey captured and predator survival, produced nearly identical results to those of the simpler model presented here.

For the simulations presented here, I set *N*_*ENC *_to 4, *P*_*C *_to 0.5, and *N*_*MIN *_to 1. The exact values of these parameters seemed not to matter much as long as they permitted the predator-prey system to persist under a stationary optimum. I also assumed that the predator population size was smaller than that of the prey. Hence, I set *K *and *B *to 256 and 4, respectively, for the prey, and to 128 and 2 for the predator. For control runs, I set *N*_*MIN *_to 0 during the experimental generations, so predators in the control replicates did not need to catch the focal prey species to survive. The results presented here assume that the traits of both predator and prey are subject to stabilizing selection of the same strength, and that their optima move at the same rate. Additional analyses indicate that these assumptions can be relaxed without changing the major conclusions of the paper. For example, if the predator's optimum does not move, then the positive effect of the predator on the extinction time of the prey still occurs but is less pronounced for prey populations that persist for long periods of time, because the predator quickly goes extinct when all of the prey evolve to be larger than the maximum gape size that can evolve under the static selection regime.

### The competition model

Interspecific competition was included in the model by adding an additional round of selection just before the reproductive phase of the life cycle. The survivors of viability selection (according to equation 1) were less likely to survive to reproduction if they possessed a trait value near the population mean of the competing species, a standard assumption in the formulation of co-evolutionary models [28,29]. This model thus assumes that the same trait that mediates the competitive interaction also responds to environmental change, as might happen for example with beak size in Darwin's finches [23,24]. The probability of perishing due to competition dropped off according to a Gaussian-shaped function with a variance equal to the trait variance in the competing species, and the maximum probability of dying as a consequence of competition was set by the competition coefficient *C*, scaled linearly by the number of individuals of the competitor present. Thus, the probability that an individual of species *i *would survive competition with species *j *was given by the equation

(3) $$W_{ij}=1-C\frac{N_{j}}{K_{j}}\left\{\exp\left[\frac{0.5{\left(z_{i}-{\bar{z}}_{j}\right)}^{2}}{\sigma_{pj}^{2}}\right]\right\},$$

where *N*_*j *_is the number of individuals of species *j *surviving viability selection, *z*_*i *_is the phenotypic value of the individual of species *i *under consideration, ${\bar{z}}_{j}$ is the phenotypic mean of species *j*, and $\sigma_{pj}^{2}$ is the phenotypic variance of species *j*. The results presented here are based on a competition model that includes both intraspecific and interspecific competition, such that species 1 competes with species 1 and with species 2 according to the above equation. Elimination of the intraspecific competition has only minor quantitative effects on the results. All parameters for both competing species were set at the same values as those of the prey in the predator-prey model, except that *B *was set at 2 to increase computational speed and *C *was set to 0.25.
